# P-746. Dalbavancin Reduces Readmissions and Nephrotoxicity in Skin and Soft Tissue Infections: Real-World Comparative Effectiveness

**DOI:** 10.1093/ofid/ofaf695.957

**Published:** 2026-01-11

**Authors:** Ali Dehghani, Joanna Henry, George Yendewa

**Affiliations:** Department of Medicine, Case Western Reserve University School of Medicine, Cleveland, OH; Department of Emergency Medicine, Case Western Reserve University School of Medicine, Cleveland, Ohio; Department of Medicine, Case Western Reserve University School of Medicine, Cleveland, OH

## Abstract

**Background:**

Dalbavancin, a long-acting lipoglycopeptide, is approved for acute bacterial skin and soft tissue infections (SSTIs). Its single-dose regimen facilitates discharge and reduces need for outpatient parenteral therapy, aiding patients with housing instability, substance use, or psychiatric illness. While trials show non-inferiority to vancomycin, real-world safety and utilization data are limited. We evaluated whether dalbavancin improves healthcare use and safety outcomes versus vancomycin, linezolid, and daptomycin in a large, matched SSTI cohort.

**Figure 1.**
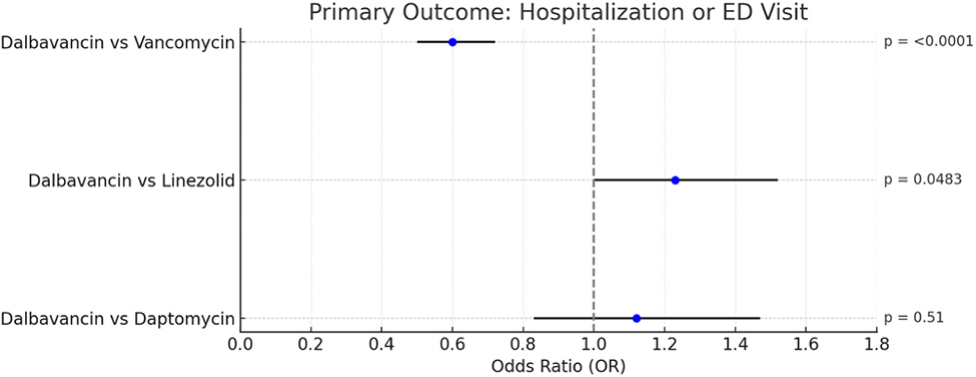
Forest Plot of Primary Outcome: Hospitalization or Emergency Department (ED) Visit Forest plot comparing odds of infection-related 90-day readmissions among patients with skin and soft tissue infections treated with dalbavancin versus vancomycin, linezolid, or daptomycin. Dalbavancin was associated with significantly reduced odds of readmission compared to vancomycin (OR 0.66), linezolid (OR 0.62), and daptomycin (OR 0.36), all with p < 0.0001. Dashed line indicates the null effect (OR = 1.0); horizontal bars represent 95% confidence intervals.

**Figure 2.**
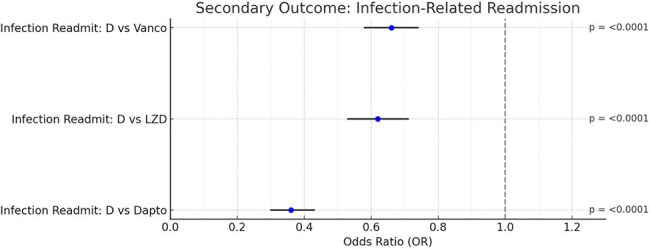
Forest Plot of Secondary Outcome: Infection-Related Readmission Forest plot comparing odds of infection-related 90-day readmissions among patients with skin and soft tissue infections treated with dalbavancin versus vancomycin, linezolid, or daptomycin. Dalbavancin was associated with significantly reduced odds of readmission compared to vancomycin (OR 0.66), linezolid (OR 0.62), and daptomycin (OR 0.36), all with p < 0.0001. Dashed line indicates the null effect (OR = 1.0); horizontal bars represent 95% confidence intervals.

**Methods:**

Using TriNetX, we performed three retrospective, 1:1 propensity-matched cohort studies comparing dalbavancin to vancomycin (n=1,052), linezolid (n=989), and daptomycin (n=487) in adults with SSTIs. Matching included age, sex, race, comorbidities (e.g., diabetes, end-stage renal disease [ESRD], HIV), substance use and mental illness. We excluded patients with osteomyelitis, bacteremia, or ICU stays within one month of the index date. Outcomes were assessed within 90 days and included hospital/emergency department (ED) visits, infection-related readmissions, acute kidney injury (AKI), drug-induced liver injury (DILI), rhabdomyolysis, and cytopenias. Odds ratios (ORs) were estimated using multivariable logistic regression models.

**Figure 3.**
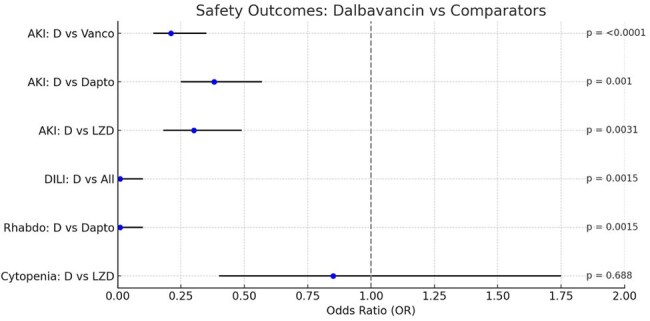
Forest Plot of Safety Outcomes: Dalbavancin vs. Comparator Antibiotics Forest plot comparing the odds of adverse safety outcomes in patients treated with dalbavancin versus vancomycin, linezolid, and daptomycin for skin and soft tissue infections. Dalbavancin was associated with significantly lower odds of acute kidney injury (AKI) compared to vancomycin (p < 0.0001), daptomycin (p = 0.001), and linezolid (p = 0.0031). Drug-induced liver injury (DILI) and rhabdomyolysis occurred exclusively in comparator groups (p = 0.0015 for each). No significant difference in cytopenia was observed between dalbavancin and linezolid (p = 0.688). Dashed vertical line indicates the null value (OR = 1.0); horizontal bars represent 95% confidence intervals.

**Results:**

Across all matched cohorts, mean age was 54 years, with 52–58% male and 74–84% White. Dalbavancin was associated with significantly lower infection-related 90-day readmission rates compared to vancomycin (29.1% vs. 38.1%; OR 0.66, p< 0.0001), linezolid (30.3% vs. 41.6%; OR 0.62, p< 0.0001), and daptomycin (27.7% vs. 51.7%; OR 0.36, p< 0.0001). Hospitalization or ED visits were also reduced versus vancomycin (27.1% vs. 38.2%; OR 0.60, p< 0.0001). Dalbavancin had significantly lower AKI rates (< 1%) compared to vancomycin (5.3%), daptomycin (8.2%), and linezolid (3.5%; all p< 0.001). DILI and rhabdomyolysis occurred only in comparator groups. No significant cytopenia differences were observed.

**Conclusion:**

Dalbavancin was associated with reduced 90-day readmissions and nephrotoxicity in real-world SSTI care. Its simplified dosing benefits patients with adherence barriers and supports value-based care models.

**Disclosures:**

All Authors: No reported disclosures

